# Two new species of *Abernessia* Arlé (Pompilidae, Ctenocerinae)

**DOI:** 10.3897/zookeys.353.6223

**Published:** 2013-11-20

**Authors:** Cecilia Waichert, James P. Pitts

**Affiliations:** 1 Utah State University, Department of Biology, 5305 Old Main Hill, Logan, UT 84322–5305, USA

**Keywords:** Ctenocerinae, Neotropical, new record, spider wasp, taxonomy, key

## Abstract

Two new species are added to the rare pompilid genus *Abernessia* Arlé. *Abernessia capixaba* **sp. n.** and *A. giga* **sp. n.** are described and illustrated. This is the first record of the genus from the states of Espírito Santo and Minas Gerais, Brazil. The genus now contains four species. A brief discussion of generic characters, illustrations, and a key to the known species of *Abernessia* are provided.

## Introduction

The spider wasps (Pompilidae) form a cosmopolitan family comprised of approximately 5,000 species and more than 120 genera (Pitts et al. 2006), with the greatest species richness in the tropical areas (Wasbauer 1995). Four subfamilies are currently recognized in Pompilidae: Ceropalinae, Ctenocerinae, Pepsinae, and Pompilinae (Pitts et al. 2006).

Ctenocerinae has 18 described genera and is found in South America, Africa, and Australia (Arnold 1932; Evans 1972; Fernández 2000; Waichert and Pitts 2011). Rodriguez (personal communication) has found the first fossil of Ctenocerinae from the Florissant Fossil Beds, Colorado, USA. This fossil indicates a wider distribution of extinct lineages of Ctenocerinae in the New World. Currently, the subfamily is diverse in the Afrotropical region, and rare in the Neotropical region (excluding *Epipompilus* Kohl). Neotropical Ctenocerinae are identified by 1) lacking subapical spine-like setae in grooves or pits on the meso and metafemur; 2) the fore wing with the Cu1 vein simple at the base; 3) the pronotum with dorsal and lateral faces; 4) the clypeus and face flattened and polished with a bilobed clypeus; 5) the face with a deep and broad antennal scrobe; 6) the scale-like setae clothing the integument; 7) and the head with a prolonged vertex (Waichert and Pitts 2011).

The Neotropical genus *Abernessia* was described by Arlé (1947) to include a single female from Rio de Janeiro, Brazil. The genus remained monotypic until Waichert and Pitts (2011) described the first male of Ctenocerinae from the Neotropics. This male was placed in *Abernessia* due to similarities in morphology, especially the flat clypeus that is undifferentiated from the face and the large antennal scrobes. Herein, we describe two species of *Abernessia* – one species based on the female sex and another based on the male sex, which is the second Neotropical record for males in the subfamily. Finally, a key for the genus and illustrations are provided.

## Material and methods

Abbreviations used in the descriptions are the same as those used by Wasbauer and Kimsey (1985). They are defined as follows: FD, the facial distance; LA3, the length of third antennal segment; MID, the middle interocular distance; OOL, the ocellocular length; POL, the postocellar length; TFD, the transfacial distance; UID, the upper interocular distance; and WA3, the width of third antennal segment. Measurements of clypeus are as follow: WC, width of clypeus, measured from the widest points; and LC, highest length of clypeus. Additional to the standard measurements, we determined the height of eye, measured in frontal view (HE).

The males described here were collected as part of the project “N.E.S.H. – Nucleus of Excellence in Systematics of Hymenoptera: broadening agricultural and environmental frontiers of Espírito Santo”, grant FAPES/CNPq #52263010/2011, coordinated by Dr. Celso O. Azevedo.

Material examined is deposited in Coleção Entomológica da Universidade Federal do Espírito Santo (UFES), Vitória, Brazil and Zoological Museum University of Copenhagen (ZMUC), Copenhagen, Denmark, as indicated.

## Systematics

### Subfamily Ctenocerinae Dahlbom

#### Genus *Abernessia* Arlé, 1947

##### Abernessia capixaba sp. n.

http://zoobank.org/D39506D7-B032-4242-9223-BC9BCA273E5E

http://species-id.net/wiki/Abernessia_capixaba

###### Holotype.

**Figures 1–3.**
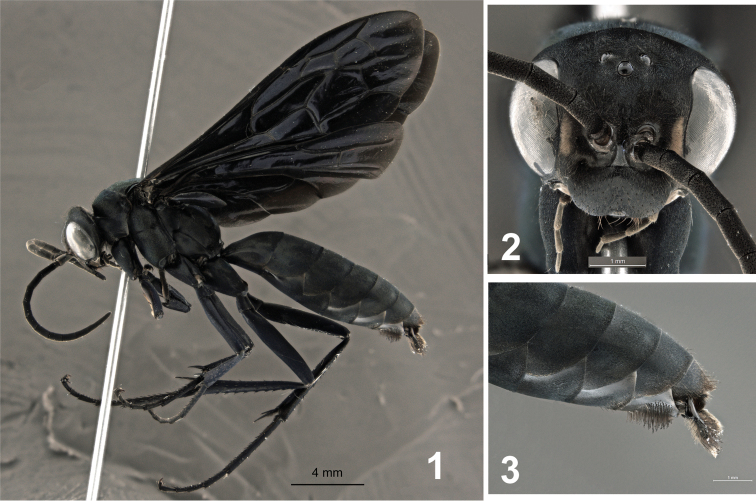
Male holotype of *Abernessia capixaba* sp. n. **1** Lateral habitus **2** Head, frontal view **3** Apex of metasoma in lateral view.

♂ (Figs 1–3), pinned, with genitalia in a separate vial, labeled “BRAZIL: E[spírito] S[anto], Laranja da Terra, Joatuba, Fazenda Betzel, 19°50'25"S, 40°49'40"W, Malaise Bosque 9, 280–430 m, 05–12.x.2012, M.T. Tavares & eq. col. (UFES #135382)”.

###### Paratypes.

2♂: BRAZIL: E[spírito] S[anto], Laranja da Terra, Joatuba, Fazenda Betzel, 19°50'25"S, 40°49'40"W, 280–430 m, 05-12.x.2012, M.T. Tavares & eq. col., Malaise Bosque 3 (1♂) (UFES #134333), Malaise Bosque 12 (1♂) (UFES #134542).

###### Diagnosis.

This species can be recognized by the following unique combination of characters: the integument is black with scale-like setae reflecting greenish metallic (Fig. 1); the face has small whitish spots on inner margin of the eyes (Fig. 2); and the wing is darkened without pale maculations (Fig. 1).

###### Description.

Body length 2.00 cm; fore wing 1.82 cm; maximum wing width 0. 57 cm.

**Coloration.** Integument black with pale yellow maculation on inner margin of eyes; body covered with pubescence with bluish-green metallic reflections (Fig. 1); clypeus, antennae, labial and maxillary palpi black; wings black with weak purple reflections; veins dark castaneous; legs with greenish-purple-blue reflections.

**Head.** Head wide; TFD 1.2 × FD; MID 0.7 × FD; punctuation conspicuous, small, shallow. Ocelli in obtuse angle; lateral ocelli closer to each other than to compound eyes; POL 0.8 × OOL. Mandible narrow, base about 2.0 × wider than apex, with two sharp apical teeth; 1/3 of base covered by thin copper pubescence. Clypeus undifferentiated from frons, flat, bilobed, apical median margin invaginated (Fig. 2); clypeal lobes rounded (Fig. 2); LC 0.4 × WC. Labrum partially exposed. Maxillary beard not present. Flagellum elongate; length of second flagellomere 2.5 × width; ratio of the scape, pedicel, and flagellomeres 1-2 11:4:14:15; WA3 0.5 × LA3; LA3 0.4 × UID; scape with erect setae on internal margin. Torulus circular, antennal scrobe large.

**Mesosoma.** Pronotum not elongated (Fig. 1), width 3.3 × length; posterior margin arched, anterior margin slightly invaginated medially; propodeal disc with thin-shallow median sulcus. Notauli shallow, present on 1/5 of anterior margin. Postnotum striated. Propodeum with punctures small, almost inconspicuous under setae; propodeal disc covered with short-apressed pubescence, setae equally abundant on propodeal disc; propodeal disc slightly elevated medially, edges of disc rounded. Wing elongate; maximum width 0.3 × length; third submarginal cell about as long as second submarginal cell; second recurrent vein straight, meeting third submarginal cell half distance from base to apex of cell (Fig. 1). Fore tibia with short, sharpened spines, posterior edge angulated. Front tarsal claw bifid, mid and fore claw dentate. Tarsi spinose.

**Metasoma.** Metasoma covered by short, scale-like setae. Sternum 7 covered by thick, long setae, marginal setae longer than remaining setae, apex of setae sinuous and dilated (Fig. 3).

**Figures 4–6.**
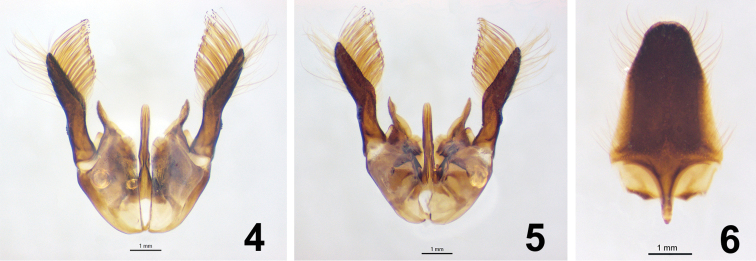
Male genitalia, paratype of *Abernessia capixaba* sp. n. **4** Dorsal view **5** Ventral view **6** Genital plate.

**Genitalia.** (Figs 4–6) Parapenial lobe bifid; lobe wide, short, almost shield-like; outer apex lanceolate, higher than inner apex; inner apex rounded, broad. Dorsal lobe of digitus slightly longer than edeagus, apex wide, rounded, with small extension ventrally; ventral lobe of digitus, short, length 0.3 × paramere length, spatulate, base with long, thin setae (Figs 4, 5). Aedeagus short, total length 0.6 × length of paramere + gonobase, split, lateral margins rolling inwards, apex rounded (Figs 4, 5). Paramere as long as aedeagus, constricted on base, wide apically; apex lanceolate, covered with long setae, inner face flat, outer rounded; setae long, longer marginally, apex dilated. Subgenital plate elongate, wide; apex narrower, rounded; apical margin of apex polished, glabrous; abundant setae, long, thin along entire length (Fig. 6).

###### Variation.

No significant morphological variation was observed.

###### Etymology.

The specific epithet refers to the type locality. *Capixaba* refers to a person born in Espírito Santo State, Brazil.

###### Remarks.

Males of *Abernessia capixaba* are distinguished from those of *Abernessia prima* Waichert & Pitts (2011) by the lack of pale maculation on the metasoma and the fully fuscous fore wing (Fig. 1). In *Abernessia prima* the fore wing is partially yellow and maculations are present on the face, metasoma, and fore wing. Finally, the setae on the subapical metasomal sternite are longer on the outer margin in *Abernessia capixaba*.

##### Abernessia giga sp. n.

http://zoobank.org/2DB96132-8EE5-498D-936A-DFF88AC8C599

http://species-id.net/wiki/Abernessia_giga

###### Holotype.

**Figures 7–9.**
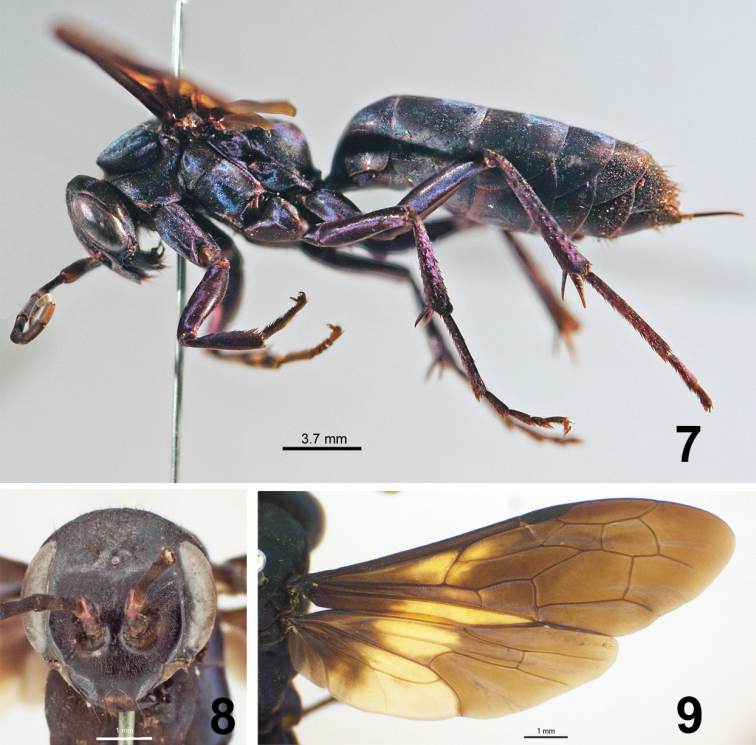
Female holotype of *Abernessia giga* sp. n. **7** Lateral habitus **8** Face, frontal view **9** Fore and hind wings.

♀ (Figs 7–9), labeled “[BRAZIL]: Minas Gerais, Reinhardt. Mus: Drenis (ZMUC)”.

###### Diagnosis.

This species can be recognized by the following unique combination of characters: the integument is black, with scale-like setae reflecting metallic bluish-green (Fig. 7); the antennal scape is red apically (Fig. 8); the clypeus is slightly folded ventrally on the apical-lateral margin, and the wing is dark with a large pale brown band (Fig. 9).

###### Description.

Body length 2.81 cm; fore wing 2.00 cm; maximum wing width 0.63 cm.

**Coloration.** Head black; mesosoma black, legs brown with purple reflections (Fig. 7); metasoma brown, tergites distally reddish, hypopygium reddish; body pubescence with bluish-green metallic reflections; wings dark brown with pale brown spots, hind wing with pale brown band (Fig. 9); wing venation brown, pale brown on pale spots; stigma brown (Fig. 9).

**Head.** Head as long as wide; TFD 1.0 × FD; MID 0.7 × FD; punctuation conspicuous, small, shallow. Pubescence abundant, short, thin, apressed, with metallic reflections from above ocelli to vertex. Eye short, HE 0.6 × FD; vertex long, distance from posterior ocellus to vertex 0.2 × FD. Ocelli in an obtuse angle; lateral ocelli closer to each other than to compound eyes; POL 0.3 × OOL. Mandible wide, elbowed, with two sharpened apical teeth; distal margin with a row of setae. Clypeus undifferentiated from frons, flat, bilobed, apical median margin invaginated; clypeal lobes rounded, sides slightly turned downwards (Fig. 8); LC 0.4 × WC. Labrum partially exposed, setose (Fig. 8). Maxillary beard not present. Flagellum elongate; length of second flagellomere 3.1 × width; ratio of the scape, pedicel, and flagellomeres 1-2 15:3:16:14; WA3 0.3 × LA3; LA3 0.4 × UID; scape curved, internal margin flat. Torulus circular, antennal scrobe large.

**Mesosoma.** Pronotum not elongate (Fig. 7), width 1.6 × length; posterior margin arched, anterior margin slightly invaginated medially; propodeal disc with thin-shallow median sulcus, lateral margins rounded. Notauli shallow, complete. Postnotum striated. Propodeum with punctures small, almost inconspicuous under setae; propodeal disc covered with short-apressed pubescence, setae equally abundant; propodeal disc slightly elevated medially, edges rounded. Wing long; maximum width 0.3 × length; third submarginal cell about as long as second submarginal cell; second recurrent vein straight, meeting third submarginal cell about half the distance from base to apex of cell; 2r-m straight (Fig. 9). Fore tibia with short, sharpened spines, posterior edge angulated. Front tarsal claw bifid, mid dentate (hind tarsi broken in holotype). Tarsi spinose.

**Metasoma.** Metasoma long (Fig. 7), total length 1.4 × mesosoma + head lengths, wide; covered by short, scale-like setae. Apical tergite setose.

###### Etymology.

The specific epithet was taken from Greek, *giga*, meaning “giant” in English. It refers to the large size of the specimen.

###### Remarks.

This species can be distinguished from *Abernessia irmgardae* Arlé by having the wing brown, with venation both pale and dark brown. In *Abernessia irmgardae* the wing is yellow and the venation is only dark brown. Additionally, the eyes are convergent on vertex in *Abernessia giga* and the vertex expanded unlike in *Abernessia irmgardae*.

##### Key to the species of *Abernessia*

###### Females

**Table:** 

1	Wings yellow, slightly darkened at the base; fore wing with pale brown vein; head with vertex weakly prolonged posteriorly; eyes not convergent on vertex	*Abernessia irmgardae* Arlé
–	Wings darkened, with spots in pale brown (Fig. 9) near the base; fore wing with venation both pale and dark brown; hind wing with a pale brown band; head with vertex strongly prolonged posteriorly; eyes convergent on vertex (Fig. 8)	*Abernessia giga* sp. n.

###### Males

**Table:** 

1	Wings pale brown near base, darkened apically; large whitish spots on inner margin of eyes, clypeus and metasoma	*Abernessia prima* Waichert & Pitts
–	Wings black with purplish reflections (Fig. 1); small whitish spots only on inner margin of eyes (Fig. 2); whitish spots absent on metasoma	*Abernessia capixaba* sp. n.

## Discussion

*Abernessia* is a species-poor genus totaling four described species. All four species of *Abernessia* appear to be restricted to Brazil. Two species have precise collecting data (*Abernessia prima* and *Abernessia capixaba*) indicating that they were collected in ecotones of the Brazilian cerrado and Atlantic forest. Additionally, this is the first record of *Abernessia* in the states of Espírito Santo and Minas Gerais and the first time that more than one specimen was collected in the same locality providing the opportunity to account for morphological variation for the group. Although males and females differ in several features, all four species of *Abernessia* are diagnosed by the following characteristics: the clypeus is flat and undifferentiated from the face; the antennal scrobe is large (Figs 2, 8); the labrum is partially exposed with setae present apically; the metasoma is longer than the mesosoma; and the pronotal disc is flat dorsally with a median sulcus on the anterior margin. Additionally, males have yellowish pale spots on the inner side of the eyes (Fig. 2); the subapical sternite and paremere have long setae that are swollen on the apex (Fig. 3); and the genitalia has a short base, which gives a false impression of long parameres (Figs 4, 5).

We were unable to associate the sexes of the newly described species. Unfortunately, the type of *Abernessia irmgardae* could not be located for morphological studies. Based on the wing and body coloration, *Abernessia capixaba* could be the male of *Abernessia irmgardae*, and *Abernessia prima* of *Abernessia giga*. However, *Abernessia capixaba* differs from *Abernessia irmgardae* by lacking reddish color on the median flagellomeres and on the apex of tarsi, and by having the wings black, whereas in *Abernessia irmgardae* the wings are yellow but dark brown basally. Additionally, the second submarginal cell is slightly different from the type by having the vein inclined downwards, while in the female type it is bent upwards. *Abernessia giga* resembles *Abernessia prima* by having pale spots on the wing; *Abernessia prima*, however, has a different pattern of wing coloration and presents whitish spots on metasoma. In other genera of Pompilidae (e.g. *Priocnemella* Banks, *Phanochilus* Banks), wing venation and coloration usually matches between sexes of a single species, even when sexual dimorphism is present. Although, wing venation in *Abernessia* seems only slightly variable, differences between specimens are obvious. Because the dimorphism sexual is not understood in the genus yet, we believe it unwise to associate these sexes prematurely.

## Supplementary Material

XML Treatment for Abernessia capixaba

XML Treatment for Abernessia giga
